# Magnetic Nanoparticles in Human Cervical Skin

**DOI:** 10.3389/fmed.2019.00123

**Published:** 2019-06-04

**Authors:** Kari Murros, Joonas Wasiljeff, Elena Macías-Sánchez, Damien Faivre, Lauri Soinne, Jussi Valtonen, Marjatta Pohja, Pekka Saari, Lauri J. Pesonen, Johanna M. Salminen

**Affiliations:** ^1^Department of Neurology, Helsinki University Hospital, Helsinki, Finland; ^2^Department of Geosciences and Geography, University of Helsinki, Helsinki, Finland; ^3^Department of Biomaterials, Max Planck Institute of Colloids and Interfaces, Potsdam, Germany; ^4^CEA, CNRS, BIAM, Aix-Marseille University, Cadarache, France; ^5^Department of Plastic Surgery, Helsinki University Hospital, Helsinki, Finland; ^6^Department of Physics, University of Helsinki, Helsinki, Finland

**Keywords:** magnetite, nanoparticles, human tissue, skin, Parkinson's disease, superparamagnetic, gut

## Abstract

Magnetic iron oxide nanoparticles, magnetite/maghemite, have been identified in human tissues, including the brain, meninges, heart, liver, and spleen. As these nanoparticles may play a role in the pathogenesis of neurodegenerative diseases, a pilot study explored the occurrence of these particles in the cervical (neck) skin of 10 patients with Parkinson's disease and 10 healthy controls. Magnetometry and transmission electron microscopy analyses revealed magnetite/maghemite nanoparticles in the skin samples of every study participant. Regarding magnetite/maghemite concentrations of the single-domain particles, no significant between-group difference was emerged. In low-temperature magnetic measurement, a magnetic anomaly at ~50 K was evident mainly in the dermal samples of the Parkinson group. This anomaly was larger than the effect related to the magnetic ordering of molecular oxygen. The temperature range of the anomaly, and the size-range of magnetite/maghemite, both refute the idea of magnetic ordering of any iron phase other than magnetite. We propose that the explanation for the finding is interaction between clusters of superparamagnetic and single-domain-sized nanoparticles. The source and significance of these particles remains speculative.

## Introduction

Magnetic analyses of human tissues have revealed the presence of nanosized ferrimagnetic magnetite/maghemite particles (MNPs) in the brain, meninges, heart, liver, and spleen (1–7). The two distinct types of magnetic crystals identified in human brain are euhedral crystals possibly of biogenic origin (1, 5, 8, 9) and rounded crystals of combustion-derived origin (5). Many organisms are able to biochemically precipitate magnetite (10), and one theory is that biogenic magnetic nanoparticles in human tissues crystallize *in situ*, with the possible precursor being ferritin (11, 12).

The magnetic properties of magnetite depend on magnetic relaxation time (13), determined by particle size, morphology, the microscopic coercive force of the particle, saturation magnetization of the material, and thermal energy (14–17). Particles with ultrashort relaxation times are referred to as superparamagnetic (SPM). For magnetite, particles under 30 nm are considered SPM, whereas those of 30–80 nm are single-domain (SD) particles (14, 17, 18). At room temperature, SD particles are able to carry magnetic remanence, while SPM are not (18). Generally, static magnetic fields can reduce mitochondrial membrane potentials, generate oxidative stress, and induce apoptotic processes (19, 20). Magnetic nanoparticles can translate possible effects to tissues. In human cells, magnetic nanoparticles have induced loss of mitochondrial membrane potential as an early sign of apoptosis (21). The magnetic iron oxide, magnetite (Fe^2+^Fe${}_{2}^{3+}$O_4_), and its oxidized equivalent maghemite (γ-Fe${}_{2}^{3+}$O_3_), may each possibly play a role in the pathogenesis of neurodegenerative diseases (3, 22). Iron and MNPs, by also containing ferric iron (Fe^3+^), can promote alpha-synuclein protein (α-Syn) aggregation (23, 24). Importantly, the α-Syn aggregates in the central and peripheral nerves are hallmarks of Parkinson's disease (PD) (25). Phosphorylated α-Syn aggregates have been detectable in cervical skin samples of PD patients but not in control samples (26). Exploring the occurrence of MNPs in skin tissue may thus reveal important information on PD pathogenesis.

The primary aim of the present study was to examine the presence and potential role of MNPs in the cervical (neck) skin of subjects with and without PD. This site was targeted because it has most consistently harbored alpha-synuclein protein pathology in PD skin samples (26). We studied the mineralogy, morphology, and composition of MNPs in human cervical skin samples by magnetometry, transmission electron microscopy (TEM), and the associated spectroscopic techniques, electron energy loss spectroscopy (EELS) and energy-dispersive X-ray spectroscopy (EDS).

## Materials and Methods

### Research Subjects and Ethical Issues

The study participants formed two groups: 10 PD patients and a control group of 10 spouses of PD patients (Table 1). The patients had an idiopathic PD meeting the UK Parkinson's Disease Society Brain Bank clinical diagnostic criteria (27). None of the controls had PD nor any signs of parkinsonism. Exclusion criteria for both groups were cognitive decline (Mini-Mental State Examination points <25), bleeding disorders, anticoagulant treatment, diabetes, dermatological abnormalities in the cervical area, and allergy to local anesthetics. This prospective study was approved by the Ethics Committee of Helsinki and the Uusimaa Health District Area of Finland, and all procedures were in accordance with relevant guidelines and regulations. Each study subject provided a written informed consent.

**Table 1:** Demographic and clinical characteristics of patients and controls.

	**Patients (*n* = 10)**	**Controls (*n* = 10)**
Age in years, median (range)	68.5 (55–74)	70.5 (50–72)
Gender, male (%)	50	50
Years from PD diagnosis, median (range)	8 (1–17)	-
Hoehn & Yahr stage, median (range)	2.5 (1.5–4.0)	-
Years with PD patient, median (range)	0	43 (1–50)
Body Mass Index, median (kg/m^2^)	26.5	24.9
Current smoking, prevalence (%)	10	20
Hypertension (medicated), prevalence (%)	40	30
Hyposmia by history, prevalence (%)	80	0

### Skin Samples

Cervical (neck) skin tissue biopsies took place at the neurological outpatient clinic of the Helsinki University Central Hospital (HUCS), Jorvi Hospital. Each skin biopsy was performed by a plastic surgeon, who cut an approximately 10-mm long boat-shaped section from the cervical skin area localized behind the right sternocleidomastoid muscle edge, anatomically corresponding to C7 spine level. The rims of the samples were removed with a knife having an aluminum shaft and a non-magnetic ceramic blade (Fine Science Tools, Heidelberg, Germany) to exclude possible contamination. Each isolated sample was packed into a non-magnetic polythene film package, and then kept in two sealed plastic bags in a freezer (−20 °C). To preclude skin-surface contamination, each skin sample, when frozen and dry, was macroscopically sectioned by the plastic surgeon with a ceramic blade into two parts: dermal (D) and epidermal (E). The D part was considered to contain some amounts of hypodermal tissue, whereas the epidermal part was a mixture of E and D tissue. The tissue samples remained in a polythene film package for room-temperature measurements and in plastic straw for low-temperature magnetic measurements. In one PD patient, the skin sample was too atrophic for proper dissection, excluding this case from the material of the present study.

### Magnetic Methods

Room-temperature magnetic measurements took place at the Solid Earth Geophysics Laboratory, University of Helsinki, with 2G (now WSGI) cryogenic superconducting quantum interference device (SQUID) magnetometry. Each analytical step was designed and monitored to exclude any possible magnetic contamination. Instrument background noise level and level of laboratory contaminants were monitored with blank 5.8-g ice cubes of distilled deionized water in an ultrasonically cleaned plastic cube kept in the laboratory environment for 4–7 days before freezing. Typical ice-cube background noise levels were in the range of 6–9 × 10^−11^ Am^2^ (intensity: 1–1.6 × 10^−8^ Am^2^ kg^−1^), corresponding with mean background noise level (6–9 × 10^−11^ Am^2^) of the instrument. To identify magnetic grain sizes and magnetic interactions, anhysteretic remanent magnetization (ARM) was induced in a decaying (from 100 mT to zero) alternating magnetic field (af), with a small, superimposed direct current field (0.05 mT), and was subsequently af-demagnetized. To study the type of magnetic material and its concentration, and to further study the grain size and magnetic interaction (28–30), we measured stepwise remanence acquisition with incremental application of direct current fields for subsets of samples (four patients, three controls), and room-temperature isothermal remanent magnetizations (IRMs). Saturation isothermal remanent magnetization (SIRM) was imparted with 3T field and subsequently af-demagnetized using the same af-steps as in ARM demagnetization. The remanence of the sample holder was subtracted from the results.

Wohlfarth's ratio (W) (28, 29) was defined from the intersection of the IRM acquisition curve and the af-demagnetization curve of SIRM. For non-interacting SD particles with uniaxial anisotropy, Wohlfarth's ratio is 0.5; lower values are attributable to particle interactions, or to SPM or multidomain (MD) influences (28, 29). The Lowrie–Fuller test (30) served for study of the domain size of magnetic grains. To distinguish between SD- and MD-sized grains, requires comparison of the coercivity spectra of the ARM and IRM. In SD grains, ARM requires larger destructive fields than does IRM to reach the same normalized remanence level. The Lowrie-Fuller test can be quantized using the parameter MDF (median destructive field). MDF is calculated as the demagnetizing field required halving the magnetization.

Low temperature magnetic measurements of skin samples were done at the Low Temperature Laboratory, Aalto University (Espoo, Finland), using reciprocating sample SQUID Magnetic Property Measurement System XL7 magnetometer (Quantum Design) for the 10 patients and 10 controls. Care was taken that the airlock valve was operated properly to prevent any air leak into the system. For low temperature analyses a straw is used as a sample holder. After the sample was mounted into magnetometer, the sample space was purged with pure He a few times, and the measurements were done at very low He pressure in the measurement chamber. An oxygen test determined that there was no leak of air into the chamber. Induced magnetization was measured as a function of temperature between 5 and 270 K in a 50 mT field after (1) the sample was cooled in a zero field (ZFC) and (2) cooled in an applied field of 50 mT (FC). In addition, induced magnetization was measured as a function of the field for one patient sample up to 1T in 48 K and in 300 K. Measured magnetic moments were normalized with the masses of completely dry tissue samples (Tables 2, 3). Each completely dry mass was weighed after the low temperature measurement. For some of the dermal samples, dry mass was calculated based on the water content of the corresponding epidermal part.

**Table 2:** Magnetic properties of dermal samples.

**Sample**	**Saturation moment (10^**−10**^ Am^**2**^)**	**Dry mass (mg)**	**Content of SD magnetite (ng/g)**	**Tp (K)**	**Tv (K)**
**PD PATIENTS**					
1D	6.22	8.0	1640.0		20
2D	20.28	10.0	4408.7		118
3D	8.04	7.0	2428.1	41	121–123
6D	51.23	4.0	27842.4		
8D	6.00	11.0	1219.2	54	120
10D	20.37	4.0	12652.2	48	121
11D	13.62	15.0	2028.0		
12D	30.54	8.0	8852.2	46	118
14D	2.67	3.0	1733.0	42	
18D	6.43	15.0	904.4	54	
Mean			6370.8		
Average deviation			6046.9		
Median			2228.1		
**CONTROLS**					
5D	8.15	8.0	2108.4		121
7D	6.90	3.0	4713.3		117
9D	3.56	8.0	943.0		
13D	9.89	13.0	1653.8		
15D	5.77	2.0	5700.6	42	123
16D	7.98	5.0	3854.1		125
17D	16.11	20.0	1759.9		115
19D	11.01	13.0	1855.4		
20D	1.89	2.0	1786.4		
21D	2.20	3.0	1913.0		
Mean			2628.8		
Average deviation			1276.3		
Median			1884.2		

*Tp, temperature for low temperature perturbation; Tv, Temperature for Verwey transition*.

**Table 3:** Magnetic properties of epidermal samples.

**Sample**	**Saturation moment (10^**−10**^ Am^**2**^)**	**Dry mass (mg)**	**Content of SD magnetite (ng/g)**	**Tp (K)**	**Tv (K)**
**PD PATIENTS**					
1E	10.84	13.9	1695.3		
2E	2.49	6.5	832.8		
3E	17.08	11.0	3375.5		
6E	8.68	8.3	2273.2		
8E	21.31	7.5	6176.8		
10E	11.70	11.2	2272.8		
11E	18.25	25.7	1543.7		
12E	14.02	7.8	3907.5		
14E	4.33	4.2	2243.3		
18E	16.46	14.7	2434.2		122
Mean			2675.5		
Average deviation			1086.7		
Median			2273.0		
**CONTROLS**					
5E	31.66	9.7	7095.5		
7E	3.87	4.5	1869.6		
9E	6.18	6.1	2203.5		
13E	11.69	14.9	1705.6		
15E	5.66	2.6	4729.1		
16E	9.94	4.6	4697.5		
17E	16.11	18.7	1872.8	42	124
19E	13.73	19.6	1522.8		
20E	16.44	6.2	5764.4		
21E	5.48	10.7	1113.8		
Mean			3257.5		
Average deviation			1815.3		
Median			2038.2		

*Tp, temperature for low temperature perturbation; Tv, Temperature for Verwey transition*.

### Magnetically Extracted Particles

The magnetic extraction procedure was done for 11 dermal samples (nine patients and two controls). Extraction for the dermal sample was based on the procedure established by Hirt et al. (7) with slight modification. Briefly, the samples were first incubated for a day in a 1-mL solution consisting of 50 μL of Proteinase K (5 mg mL^−1^) + 950 μL HEPES Buffer (50 mM). The samples were then centrifuged at 14,000 rpm for 10 min. The supernatant was discarded and the pellet was resuspended in a 1% Triton solution for another day before the preparation of TEM grids. After the initial trials, we decided to avoid magnetic separation, which failed to successfully separate magnetic particles from other nanoparticulate components.

### Transmission Electron Microscopy (TEM), Electron Energy Loss Spectroscopy (EELS) and Energy-Dispersive X-Ray Spectroscopy (EDS)

TEM measurements took place in a double Cs-corrected Jeol JEM-ARM200F, equipped with a GIF Quantum Energy Filter (Gatan) and a silicon-drift energy-dispersive X-ray spectroscopy detector (Jeol). All spectroscopy measurements were done in STEM mode. EELS spectra were recorded using a dispersion of 0.1 eV/channel, with a 5-mm spectrometer entrance aperture, a convergence semi-angle α = 20 mrads and a collection semi-angle β = 24 mrads. The energy resolution was 1.2 eV when measured at the full width at half maximum (FWHM) of the zero-loss peak. Due to rapid hydrocarbon deposition, only point measurements were chosen. Spectra presented here come from single particles, with an exposure time of 1 s and 60 frames summed (total integration time 60 s); no smoothing was applied. Background was subtracted before each edge by use of power-law fitting, and plural scattering was removed by a Fourier-ratio deconvolution, both available in Digital Micrograph 3.1 software (Gatan Inc.). Plotting of the data and normalization were carried out with OriginPro 8.5 software. For energy-dispersive X-ray spectroscopy, the specimen was tilted toward the detector (α-tilt = 18 °) to increase detection efficiency. Acquisition and analysis of the data were completed with Analysis Station software (Jeol).

### Statistics

Study of statistical distributions of the measurements, as well as the between-group analysis with non-parametric comparison (Mann-Whitney U) and correlation (Spearman R) were carried out with Statistica 13.1 software (Dell Inc., Tulsa, OK, USA).

## Results

**Figure 1:**
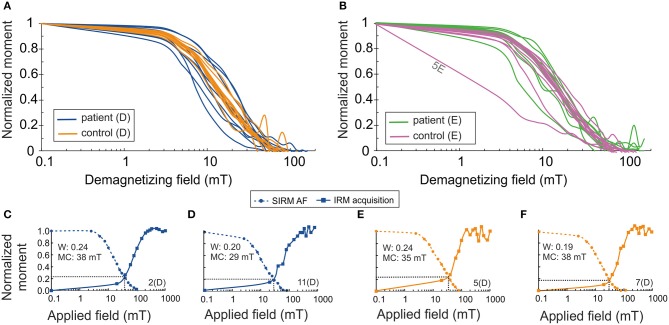
Normalized saturation isothermal remanent magnetization (SIRM/SIRM_0_) as a function of demagnetizing AF field (semi-log) **(A)** for patient samples, **(B)** for control samples. Normalized acquisition of isothermal remanent magnetization (IRM/IRM_1T_) and normalized SIRM (SIRM/SIRM_0_) as a function of AF field for cervical skin samples of patients with PD **(C,D)** and for healthy controls **(E,F)**. Wohlfarth's ratio (W) is defined from the intersection of these curves. MC, median coercivity; D, dermal; E, epidermal.

**Figure 2:**
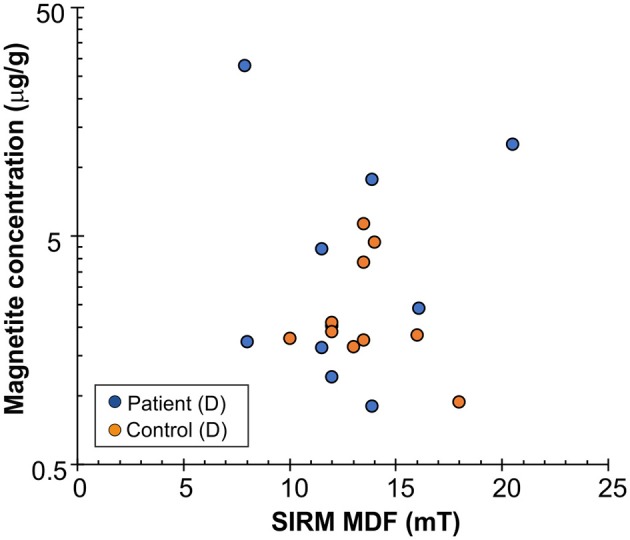
Estimated magnetite concentration (micrograms per gram, μg/g) vs. median destructive field (MDF) of saturation isothermal remanent magnetization (SIRM) for dermal part of the cervical skin samples. Y-axis in logarithmic scale.

Alternating field (af) demagnetization of SIRM of the samples supports the presence of ferrimagnetic remanence carriers (Figures 1A,B). IRM acquisition curves showed that the skin tissue samples contained low-coercivity magnetic particles which were magnetically saturated by 300 mT, indicating ferrimagnetic magnetite/maghemite (Figures 1C–F). Wohlfarth's ratios (28, 29) between 0.19 and 0.24 for both PDs and controls were obtained (Figure 1). The MDF of SIRM for PD samples showed a larger range (7.9–20.5 mT) than did those for control samples (10–18 mT) (Figures 1, 2), indicating a wider range of domain size of remanence carriers for PD samples than for controls. The limited number of subjects did not, however, provide sufficient statistical power to detect a significant difference. The Lowrie-Fuller (30) test, where MDF values of ARM are higher than those of IRM, indicates that the SD size MNPs dominate over MD size in all of the samples.

The concentration of magnetic material in the samples was calculated, assuming that the material was magnetite, having saturation magnetization 92 Am^2^/kg (1). The highest observed values of magnetite content were in the PD group, which displayed a higher spread of the distribution, most clearly in the dermal (D) samples (median 2228.1 ng/g, range 904-27842 ng/g; in controls, median 1884.2 ng/g, range 943–5,700 ng/g) (Figure 2 and Table 2). Despite the trend, no significant difference appeared between the subpopulations (*p* = 0.34, Mann-Whitney *U*-test). Values of the dermal samples showed no gender difference (*p* = 0.12, Mann-Whitney *U*-test) nor did they correlate with age or body mass index of study participants (*R* = −0.01, *p* = 0.97, and *R* = −0.32, *p* = 0.17, respectively, Spearman Rank Correlation).

**Figure 3:**
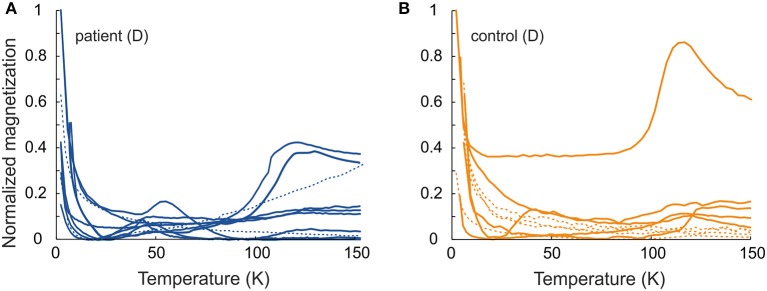
Normalized induced magnetization as a function of temperature for ZFC (M_zfc_/M_zfcmax_) **(A)** patient samples and **(B)** control samples. Solid line–ca. 50 K anomaly or Verwey transition; dashed line–no low temperature features. For comparison, the magnetization values are normalized to maximum value of ZFC magnetization.

Induced magnetization, after both low-temperature experiments, where the sample was cooled in a zero field (ZFC) and cooled in an applied field of 50 mT (FC), showed a rapid loss in magnetization on rewarming from 5 to 25 K. This range includes the temperature range for a paramagnetic signal from blood in tissue (2–8 K) (7, 31). With further warming, the ZFC showed an anomaly for the majority (six of ten) of the D samples of PD patients, and for one D sample and one E sample of the controls, with the peak ranging from 41 to 54 K (Tables 2, 3 and Figure 3). As to dermal samples, Fisher's exact test showed a trend-like difference in the occurrence of this anomaly between patients and controls (*p* = 0.06). The anomaly was absent for the FC curve. With continued warming, ZFC curves indicated Verwey transition for D samples of six PD patients at a temperature range of 118–123 K and for five D samples of controls at a temperature range of 115–125 K (Figure 3 and Table 2).

**Figure 4:**
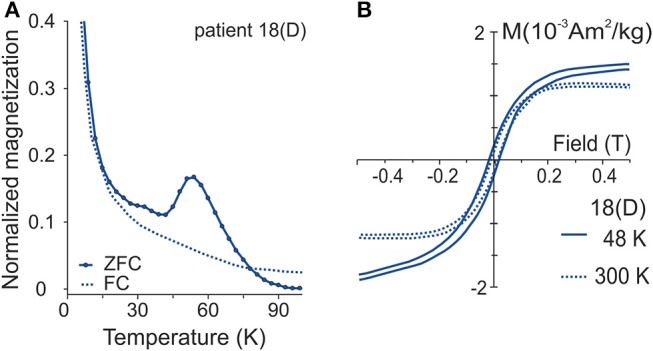
**(A)** Normalized ZFC (M_zfc_/M_zfcmax_) and FC (M_fc_/M_fcmax_) curves for patient sample 18D exhibiting 50 K perturbation. **(B)** Induced magnetization as a function of field near the anomaly at 48 K and in 300 K after slope correction. Normalization of ZFC-FC as in Figure 3.

We measured induced magnetization as a function of the field for PD patient 18D near the low temperature anomaly peak at 48 K and at 300 K (Figure 4). After the diamagnetic correction, nearly saturated magnetization emerged at both temperatures, indicating ferrimagnetic material. A slightly higher coercivity value of 20 mT appeared at 48 K, compared to 15 mT at 300 K. At 48 K, in the descending and ascending limbs of the curve in both positive and negative fields showed a bifurcation. At 300 K, this behavior was absent.

**Figure 5:**
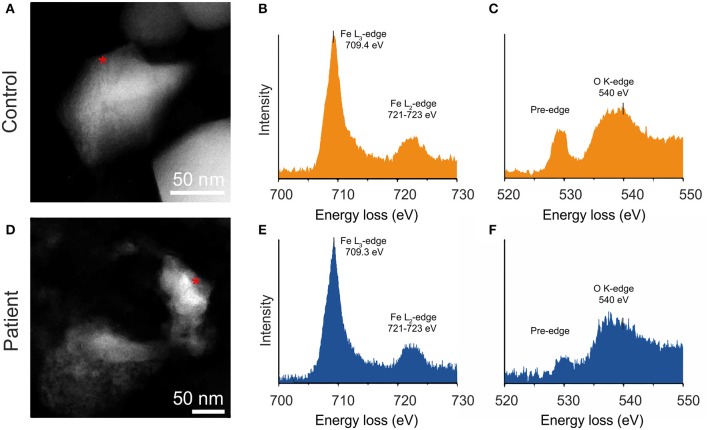
High annular dark field (HAADF) image of particles extracted from human cervical skin with respective EEL spectra; **A–C** (Healthy control), **D–F** (PD patient). Iron L_2.3_-edge **(B,E)** and oxygen K-edge fine structure **(C,F)** confirm that particles are composed of magnetite.

TEM and EELS analyses of the extracted particles confirmed the occurrence of magnetite (Figure 5). The size of these particles varied in the range of 50–100 nm, close to SD size, exceeding the size of nanoparticles formed within an 8-nm diameter of the ferritin cors (10). The nanoparticles displayed a faceted morphology, in contrast to the rounded or spherical morphologies found in brain tissue and attributed to combustion-derived sources (5). Energy loss near ege structure (ELNES) analysis of the Fe L2,3-edge clearly shows a magnetite profile, both in patients and controls (Figure 5). The L3–edge presents a single maximum located at 709.4 (±0.1) eV, and L2–edge at 721–723 eV. The energy between the two lines being 13.2 eV and the relative intensity 4.0, are in agreement with earlier values for magnetite particles (32). The O K-edge shows a pre-peak around 530 eV that presents an asymmetry, with a shoulder located at 530 eV on the high-energy side, and a principal feature at 540 eV that is common for all iron-oxide phases.

## Discussion

Room-temperature IRM and SIRM measurements identified ferrimagnetic particles in all of the samples. The broad range of Verwey transition temperatures (PD: 118–123 K; controls: 115–125 K) indicates both pure magnetite and partial oxidation of magnetite to maghemite or the presence of impurities (33), such as Ti^4+^, Zn^2+^, Al^3+^, in magnetite. TEM observations demonstrated that some of the particles were pure magnetite (Figure 5). Lowrie-Fuller test (30) refutes the presence of MD grains and Wohlfarth's ratios < 0.5 for both PDs and controls indicate that the grains capable of carrying remanent magnetization at room temperature are magnetically interacting (28, 29). The median MNP concentrations for the dry skin-tissue samples were in the same range as reported for dry brain-tissue samples (5). Although the highest concentrations of MNPs came from the PD patients, no statistical difference between PD patients and controls emerged, possibly due to the small number of samples studied.

The majority of the D samples from PD patients showed anomalous behavior in the direct current magnetization at around 50 K. Earlier low temperature measurements have indicated that heme-iron and ferritin do not contribute to the obtained anomaly around 50 K (7, 31). The only iron phase in human tissue, one that has shown anomalous behavior in its magnetic properties around 50 K, is magnetite/maghemite (34, 35). An anomaly at ca. 50 K in measurement of magnetic after-effect (MAE) or AC susceptibility has been associated with the onset of electron hopping upon warming and ionic ordering within domain walls, which indicate wall movement (36). However, the size of MNPs in this study indicates SD or SPM grain size, contradicting the presence of domain walls, being in line with the size range of MNPs in human brain tissue (7).

In 1998, Moskowitz et al. associated a low-temperature anomaly during ZFC analyses with SPM- to SD-sized maghemite particles (partly aggregated clusters) in magnetoferritin (37). Accordingly, we propose that the ~50 K anomaly indicates magnetically interacting clusters of possible SPM- and SD- sized ferrimagnetic MNPs. The peak for this perturbation occurred only on the ZFC curve, since, due to increased temperature, initially SD sized particles progressively unblock, align their moments with the applied field, and eventually randomize due to thermal energy. This anomaly did not occur during the FC analyses, since magnetization was already aligned with the field during the cooling through blocking temperature and this experiment only shows the demagnetization of the blocked particles.

Additional support for SPM grains comes from low temperature hysteresis measurements, which show higher coercivity at 48 K than at room temperature (Figure 4B), indicating the presence of SPM grains as being able to carry remanence at 48 K (4). The bifurcations similar to those obtained in the hysteresis loop have been evident in studies on human tissue (7, 36), with a suggestion that the switching of the a-b crystalline axes of magnetite around 45 K could possibly explain this bifurcation (36). In the initial field, the easy axis of magnetization would lie along one of the axes. As the field is reduced and then raised to the starting value again, what may occur is a switch in preferred axis, leading to the bifurcation observed.

Another explanation for the anomaly at ~50 K may be the magnetic ordering of molecular oxygen, which undergoes an antiferromagnetic transition at about 43 K being strongly paramagnetic above this temperature (38). We took care to prevent any oxygen leak into the system. Furthermore, the magnetic intensity of the ~50 K signal was for most of the samples an order of magnitude larger (10^−4^ Am^2^/kg) than known effects related to the magnetic ordering of oxygen at 43 K (< 2 × 10^−5^ Am^2^/kg) (7), providing additional evidence against the oxygen as a cause for the ~50 K anomaly.

Our magnetometric studies revealed MNPs with SD characteristics in all skin samples and interacting SPM characteristics for the majority of the D samples from the PD patients. The shapes of the SD-size magnetic nanoparticles studied by TEM did not resemble the cubo-octahedral crystals of possible biogenic magnetite (1, 9), but merely resembled non-biogenic magnetite. No rounded or spherical morphologies were detectable. EEL spectra of four samples demonstrated that their particles were composed of magnetite (Figure 5); on the basis of their size, we propose a source different from that of ferritin cores.

The origin of MNPs in human tissues has remained speculative (biogenic, non-biogenic, or anthropogenic/combustion). Polluted air (combustion-derived MNPs) via the respiratory route has been proposed as one source of these MNPs (5). The olfactory route was considered to be the passageway to the brain for these round and spherical particles (5), but was recently contradicted (6). Here, no particles were detectable with such morphology. However, we cannot fully exclude that airborne MNPs, via a respiratory route or even through skin structures, may in part explain the results. Low-temperature magnetic measurements showed the presence of apparent SPM particles mainly in the dermal samples of PD patients (Tables 2, 3), hinting strongly of a source other than the skin surface. As to intrinsic sources, the ferrihydrite core of the iron-storage protein ferritin has been proposed to be a precursor of MNPs (11, 12). Considerable argumentation goes against this view, as the size of single-domain MNPs is not compatible with the 6- to 8-nm diameter of the inner cavity of the ferritin protein (10). Further, nuclear magnetic resonance relaxometry studies have shown that the content of magnetite in the ferrihydrite core is < 1% (39). At least, what is not ruled out is that ferritin could by some mechanism produce small superparamagnetic MNPs, which can then aggregate into larger MNPs with SD-like characteristics. Experimens have shown that nucleation and growth of magnetite proceeds through rapid agglomeration of nanosized primary particles (40). Recently, it has been reported that human stem cells have an ability to synthesize magnetic nanoparticles from nanodegeneration products (41).

A potential source of MNPs may be the gastrointestinal tract. Having been present in many organisms (10), MNPs may be expected to be found in various foodstuffs, which, when ingested, are probably absorbed into the bloodstream from the gastrointestinal tract. In addition, drinking-water can contain MNPs (42). Ferrihydrite in soils deserves attention, since in its amorphous form, it can percolate into groundwaters and further into drinking-water (43), and magnetite crystal formation may occur by an abiotic mechanism (44). As to bacteria sporadically residing in the human gastrointestinal tract, sulfate-reducing *Desulfovibrio* species are able to synthesize magnetite (45–47). As these bacteria also produce hydrogen sulfide, known to cause olfactory dysfunction (48), a prevalent phenomenon in PD (49), the potential occurrence of these bacteria in PD is relevant. The uptake of nanoparticles from the intestinal lumen into the blood circulation may take place mainly through an endocytic transcellular transport, with even 100-nm-sized particles transported (50). Iron nanoparticles can evidently cross plasma membranes by a non-endocytic pathway, gaining direct access to the cytoplasm (51). The gut epithelial enteroendocrine cells, known to contain α-Syn, may therefore be structures to display α-Syn aggregation promoted by magnetite nanoparticles (24, 52, 53). In the blood, nanoparticles become coated by proteins, small molecules and ions (50). Tissue macrophages are considered the primary cell types that phagocytose nanoparticles which have ended up in the blood circulation (54). As the macrophage density in normal human dermis is considerable (55), skin may play an important role in removing MNPs from the blood circulation. This view is supported by an animal study showing intravenously administered nanoparticles to accumulate in the dermis, first localizing in dermal macrophages (56). The substantial magnetite content of the skin samples detected in this study may reflect such a process.

MNPs can evidently interfere with cell functions. Interactions of SPM particles with prolonged external magnetic fields may lead to biological impacts, because SPM particles have a strong magnetic susceptibility; relatively weak static magnetic fields can induce loss of membrane potentials in mitochondria (19, 20), which may potentially lead to mitophagy (56). Magnetite nanoparticles *per se* may induce mitochondrial dysfunction (21), and promote, in an uncoated form, α-Syn aggregation (24).

In the present pilot study, the number of samples was too small to allow definitive conclusions as to the true differences in MNP characteristics and quantities between the study groups. As an additional factor, PD is a disease with diverse characteristics in which various pathophysiological mechanisms may come into play (57), and the overall accuracy of clinical diagnosis of PD is far from satisfactory particularly in its early stages (58, 59). Notably, relatively high amounts of single-domain MNPs were observable in all cervical skin samples. A possible clustering of such particles with superparamagnetic particles needs further exploration in Parkinson's disease. Our study raises questions as to the origin and role of MNPs in physiologic and pathologic states in human beings as well as to the importance of the skin as an internalizer of these particles.

## Data Availability

The datasets generated for this study are available on request to the corresponding author JS.

## Ethics Statement

Ethics Committee of Helsinki and Uusimaa Health District Area of Finland.

## Author Contributions

KM and JS contributed equally to the work in this research. KM, JS, LP, DF, and EM-S designed the research. KM conceived the original idea. KM, JS, JW, JV, DF, EM-S, LS, MP, and PS performed research. JS, KM, JW, LS, LP, DF, and EM-S analyzed the data. KM, JS, and JW wrote the manuscript, including input from EM-S, DF, LS, and LP.

## Contribution to the Field

Human internal organs have been reported to harbor magnetic nanoparticles. In 1992, Kirschvink and his colleagues were the first to report magnetite nanoparticles in human brain tissue. Later, apparent magnetite and maghemite particles were found in the human heart, spleen, and liver. With their magnetic properties, these iron oxide particles may have the propensity to induce deleterious effects in cells and play a role in the pathogenesis of neurodegenerative diseases. The present study, using magnetometric and electron microcopy methodology, revealed ferrimagnetic single-domain-sized magnetite/maghemite nanoparticles in all skin samples of patients with Parkinson's disease as well as in those of healthy controls, and revealed superparamagnetic material in several samples. These findings complement studies focused on magnetic material in human tissues. The particles are proposed to be airborne or gut-mediated, and the skin may play a role in internalizing them from the blood circulation. The present findings open a new perspective on the occurrence of magnetic nanoparticles in human tissues and underline the need to clarify not only the origin of these particles but also their role in physiologic and pathologic states. To this end, the skin offers a convenient target.

### Conflict of Interest Statement

The authors declare that the research was conducted in the absence of any commercial or financial relationships that could be construed as a potential conflict of interest.
